# 

**Published:** 1880-11

**Authors:** 


					﻿NOVEMBER, 1880.
TWENTY-SEVENTH YEAR.
HALL’S
Journal of Health.
E. H. GIBBS, A.M., M.D., Editor,
No, 141 EIGHTH STREET (near Broadway) NEW YORK.
Terms, $1.50 a Year, or $1.00 for Eight Months. Postage paid
				

